# Development of RALA-Based Mannosylated Nanocarriers for Targeted Delivery of Minicircle DNA Vaccines Encoding HPV-16 Oncogenes

**DOI:** 10.3390/vaccines14010018

**Published:** 2025-12-23

**Authors:** Andressa Giusti, Dalinda Eusébio, Matilde Costa, Inês Silveira, Swati Biswas, Diana Costa, Ângela Sousa

**Affiliations:** 1 RISE-Health, Department of Medical Sciences, Faculty of Health Sciences, University of Beira Interior, Av. Infante D. Henrique, 6200-506 Covilhã, Portugal; andressa.giusti@ubi.pt (A.G.); dalinda.eusebio@ubi.pt (D.E.); matilde.costa@ubi.pt (M.C.); dcosta@fcsaude.ubi.pt (D.C.); 2 Nanomedicine Research Laboratory, Department of Pharmacy, Birla Institute of Technology and Science-Pilani, Hyderabad Campus, Jawahar Nagar, Medchal, Hyderabad 500078, India; swati.biswas@hyderabad.bits-pilani.ac.in

**Keywords:** antigen-presenting cells, cervical cancer, mannose, minicircle DNA vector, RALA peptide, octa-arginine, targeted delivery

## Abstract

Background/Objectives: Cervical cancer is a leading cause of cancer-related mortality among women, primarily driven by persistent infections with high-risk human papillomavirus (HPV), particularly HPV-16. Vaccines based on plasmid DNA encoding the viral oncogenes E6 and E7 represent a promising immunotherapeutic strategy, but their efficacy remains limited due to poor cellular uptake. Cell-penetrating peptides such as RALA improve intracellular delivery, and functionalization with octa-arginine peptide conjugated to mannose (R8M) further enhances targeting of antigen-presenting cells (APCs). This study aimed to obtain the minicircle DNA (mcDNA) encoding mutant HPV-16 E6 and/or E7 antigens, and optimize its complexation with mannosylated RALA-based nanoparticles to improve vector delivery and consequently antigen presentation. Methods: Nanoparticles were formulated at different concentrations of RALA, with and without R8M functionalization. Their characterization included hydrodynamic diameter, polydispersity index, zeta potential, complexation efficiency (CE), stability, morphology, and Fourier-Transform Infrared Spectroscopy. In vitro assays in JAWS II dendritic cells (DCs) assessed biocompatibility, transfection efficiency and target gene expression. Results: Optimal conditions were obtained at 72.5 µg/mL of RALA, producing nanoparticles smaller than 150 nm with high CE (>97%) and uniform size distribution. Functionalization with R8M at 58 µg/mL preserved these characteristics when complexed with all mcDNA vectors. The formulations were biocompatible and effectively transfected DCs. Mannosylated formulations enhanced antigenic expression compared to non-mannosylated counterparts, evidencing a mannose-receptor-mediated uptake, while increasing the production of pro-inflammatory cytokines. Conclusions: Nanoparticles based on the RALA peptide and functionalized with R8M significantly improved mcDNA transfection and gene expression in APCs. These findings support further investigation of this system as a targeted DNA vector delivery platform against HPV-16.

## 1. Introduction

Cervical cancer is one of the most prevalent genital malignancies and remains one of the leading causes of cancer-related deaths among women globally [1,2]. The burden of this disease remains disproportionately high in low- and middle-income countries (LMICs), where gaps in prevention access, poor healthcare infrastructure, and insufficient treatment options contribute to the high incidence and mortality rates [2,3,4]. A significant majority of cervical cancer cases (over 90%) are caused by persistent infection with high-risk strains of human papillomavirus (hrHPV), a very common sexually transmitted virus with oncogenic potential, mainly due to the E6 and E7 oncogenes present in its genome [1,2,5,6]. Globally, HPV-16 and HPV-18 are responsible for approximately 70% of cervical cancer cases, with HPV-16 being the most prevalent [1,3,7,8]. The E6 oncoprotein contributes to carcinogenesis by disrupting apoptosis pathways, mainly by degrading the p53 tumor suppressor [3,8,9]. Equally, E7 oncoprotein plays a central role in cell cycle dysregulation, promoting uncontrolled proliferation by targeting tumor suppressor proteins, such as retinoblastoma protein (pRB), and interacting with transcription factors that keep infected cells in the S phase with persistent proliferation [3,5,8,9].

Although preventive measures are available, such as vaccination against hrHPV and early detection screening methods to prevent infection and cancer progression, they are not fully implemented in low-resource settings, mainly due to high costs and the need for specialized equipment and trained personnel. Moreover, prophylactic vaccines do not offer any therapeutic effect [1,3,8,10,11,12]. Once cervical cancer becomes invasive and is not treated in its early stages, treatment options such as surgery and chemoradiotherapy become more aggressive, carry risks of severe long-term complications, and lack specificity, with poor outcomes, especially in later-stage disease [5,13]. These limitations emphasize the urgent need for safer, more effective, and targeted therapeutic strategies to improve patient outcomes and reduce treatment-related morbidity [4].

Emerging approaches in cervical cancer therapy include DNA vaccines, a revolutionary advancement in immunization that represents a promising alternative to traditional vaccine platforms [14]. Due to their safety, adaptability, rapid production, cost-effectiveness, and storage stability, DNA vaccines are particularly attractive for large-scale immunization efforts and cost reduction in LMICs [14,15]. Furthermore, DNA vaccines can not only prevent infection but also have therapeutic effects, as they generate cellular and humoral immune responses following antigen presentation by antigen-presenting cells (APCs) [5,14,16,17,18,19].

The DNA vaccines usually employ plasmid DNA (pDNA) vectors to deliver target genes to APCs and trigger an immune response. However, pDNA vectors can present some limitations, including the induction of inflammatory reactions or antibiotic resistance in the patient, due to the presence of prokaryotic sequences in the pDNA backbone required for their production in the bacterial host [16,19,20,21]. To overcome these issues, minicircle DNA (mcDNA) represents an innovative approach because it is devoid of bacterial sequences that were eliminated through precursor plasmid-specific recombination, leaving only the essential components necessary for antigen expression in eukaryotic cells. The resultant mcDNA is a DNA vector simpler, smaller and safer than pDNA, and although it may present only modest improvements in transfection and gene expression in some contexts, its main advantage lies in its enhanced safety profile, making mcDNA an even more promising candidate for vaccines [16,19,20,21].

However, regardless of the vector used, challenges remain in achieving effective targeted delivery, cellular uptake, and transfection efficiency. To address these issues, the involvement of efficient delivery systems is essential. These systems must be complex and protect the DNA from degradation, facilitate cellular uptake, enable endosomal escape, and promote nuclear transport, while minimizing toxicity. The efficiency of DNA vaccine delivery is crucial for achieving strong immunogenicity, as the DNA must reach immune cells, particularly professional APCs [14,16,18,22,23]. Non-viral delivery systems, including cell-penetrating peptides (CPPs), have shown great potential in the field of nanotechnology owing to their customizable properties, improved safety, easy scalability, cost-effectiveness, and lower risk of immune responses compared to other materials or viral vectors [14]. For instance, glutamic acid-rich GALA and lysine-rich KALA peptides have been used in effective nucleic acid delivery, demonstrating the adaptability of CPPs to different therapeutic applications [16,24]. The cationic amphipathic peptide RALA, a modified version of the KALA peptide, was designed to enhance the efficiency of nucleic acid delivery. Its structure consists of repeating arginine, leucine, and alanine units, which provide amphiphilic and cationic properties. By replacing lysine with arginine residues, RALA achieves a higher affinity for nucleic acids. In addition to the advantages described above, this peptide also offers high transfection efficiency and versatility for functionalization with specific ligands, making it suitable for both in vitro and in vivo applications [14,25,26]. The octa-arginine (R8) peptide, composed of eight arginine residues, is a highly effective CPP in assisting the transport of various biomolecules, including nucleic acids, across cellular membranes. Its versatility supports the optimization of targeted delivery systems. For instance, functionalizing delivery systems with mannose ligands can enhance the targeted delivery of cargo to APCs, which overexpress various types of mannose receptors [16,27]. Mannose targets C-type lectin receptors on the surface of APCs, facilitating receptor-mediated endocytosis and selective uptake [28,29]. This strategy has proven effective in enhancing transfection efficiency and improving specificity in various delivery platforms [30,31,32].

Thus, the present study is focused on the development of a peptide-based nanocarrier system to improve the targeted delivery of an mcDNA vector encoding HPV-16 E6 and E7 antigens. To address the limitations typically associated with DNA vaccine delivery, such as poor cellular uptake and limited targeting, NPs were engineered using the RALA peptide and functionalized with R8-mannose conjugated (R8M), aiming to enhance transfection efficiency and direct the complexes toward APCs. After the optimization of the mcDNA complexation, the resultant NPs were subsequently characterized in terms of their physicochemical properties, including particle size, surface charge, homogeneity, DNA complexation, and complex stability. Functional performance was assessed through in vitro transfection studies using Dendritic cells (DC) to confirm the uptake and expression efficiency. Overall, this work presents a safe and effective peptide-based targeted delivery strategy with potential to improve the specificity and performance of DNA vaccines towards HPV-related malignancies.

## 2. Materials and Methods

### 2.1. Production and Purification of mcDNA Vectors

Three mcDNA vectors were generated through in vivo recombination of parental plasmids (PP) carrying the genes of interest (E6mut and/or E7mut alone or combined). These plasmids were initially derived from pMC.CMV-MCS-EF1-GFP-SV40PolyA DNA vector (System Biosciences, Palo Alto, CA, USA), and further details of the constructs are provided in the Supplementary Materials (Figures S1 and S2). The vector amplification was performed as described by our research group [33]. Briefly, bacteria were grown on Luria–Bertani (LB) agar with kanamycin (50 µg/mL) and incubated overnight at 37 °C. The colonies were then transferred to 125 mL of sterile Terrific Broth (TB) medium (20 g/L tryptone, 24 g/L yeast extract, 4 mL/L glycerol, 0.017 M KH_2_PO_4_, and 0.072 M K_2_HPO_4_, supplemented with 50 µg/mL of kanamycin, pH 7.0), and incubated in an orbital shaker (Agitorb Aralab^®^ 200, Albarraque, Portugal) at 42 °C and 250 rpm, until the optical density at 600 nm reached approximately 5. Cells were harvested by centrifugation at room temperature under aseptic conditions (2000× *g*, 20 min), and the pellet was resuspended and transferred to 125 mL of the induction medium without antibiotics (LB 25 g/L, 0.04 M NaOH and 0.01% L-arabinose, pH 7.0). Plasmid recombination was induced at 30 °C with agitation at 250 rpm for 1 h. Finally, cells were harvested by centrifugation (3900× *g*, 10 min, 4 °C), and the pellets were stored at −20 °C. For the mcDNA extraction from bacterial host pellets, a modified alkaline lysis method was performed as described by Diogo and collaborators [34]. Purification of mcDNA was carried out by loading the extracted supernatants in a Sephacryl S-1000 SF gel filtration column, coupled with the ÄKTA Pure chromatography system (GE Healthcare Biosciences, Danderyd, Sweden), as described by Almeida and collaborators [35]. The resultant fractions were concentrated using Vivaspin 6^®^ centrifugal filters of 10 KDa (Sartorius, Göttingen, Germany) and analyzed by agarose gel electrophoresis.

### 2.2. Agarose Gel Electrophoresis

Electrophoresis was performed for 40 min at 120 V using 0.8 or 1% (*w*/*v*) agarose gels in 1× TAE buffer (40 mM Tris base, 20 mM acetic acid, 1 mM EDTA, pH 8.0). GreenSafe Premium (NZYTech, Lisbon, Portugal) was added as a staining dye at a concentration of 0.012 μL/mL of TAE buffer. The molecular weight marker used was the GRS Ladder 1 kb (GRISP, Porto, Portugal). The DNA or RNA bands on the gel were visualized using a Uvitec Fire-Reader system (Uvitec Limited, Cambridge, UK).

### 2.3. Formulation and Functionalization of RALA-Based Nanoparticles

The RALA peptide (N-WEARLARALARALARHLARALARALRACEA-C), synthetized by NZYTech (Lisbon, Portugal), was reconstituted with ultrapure-grade water to a final concentration of 0.5 mg/mL. The R8M peptide, synthetized and characterized by Professor Swati Biswas (Department of Pharmacy, Nanomedicine Research Laboratory, Birla Institute of Technology & Science Pilani), as previously described [30], was also reconstituted in ultrapure-grade water to a final concentration of 0.5 mg/mL. Nanoparticles (NPs) were formulated with all three mcDNA vectors, either as binary systems (mcDNA/RALA) or ternary systems (mcDNA/RALA/R8M). An initial screening of RALA concentrations (29, 58, 87 and 116 µg/mL) was performed to complex with 2 µg of mcDNA-E7mut, and the inclusion of 58 µg/mL of R8M concentration. To formulate the NPs, 50 µL of the prepared RALA working solutions were added dropwise to a 200 µL solution containing water and mcDNA, under constant vortex agitation for 1 min. For the ternary system, 50 µL of R8M was added after the addition step of RALA peptide, under vortex agitation for 1 min. The NPs were incubated at room temperature for 30 min to allow the formation of the nanocomplexes.

### 2.4. Characterization of Nanoparticles

#### 2.4.1. Characterization of Hydrodynamic Diameter, Polydispersity Index and Surface Charge

The hydrodynamic diameter, polydispersity index (PDI) and surface charge of the mcDNA/RALA and mcDNA/RALA/R8M NPs were determined by Dynamic Light Scattering (DLS) using a Zetasizer Nano ZS analyzer (Malvern Instruments, Malvern, Worcestershire, UK) equipped with a He-Ne laser. The hydrodynamic diameter and PDI were analyzed immediately after 30 min incubation. The zeta potential was evaluated after NPs centrifugation at 10,000× *g* for 20 min at 4 °C and pellet resuspension in 1 mL of ultrapure-grade water. All analyses were conducted in triplicate at 25 °C using Zetasizer Nano software v7.03 (Malvern Instruments, Malvern, Worcestershire, UK).

#### 2.4.2. Evaluation of Complexation Efficiency

The DNA complexation efficiency (CE) in RALA NPs was evaluated by analyzing the supernatant obtained after the NP formation and centrifugation. The supernatant was injected into the agarose gel electrophoresis to detect uncomplexed DNA and analyzed using the iQuant dsDNA HS Assay kit (ABP Biosciences, Rockville, MD, USA), according to the manufacturer’s instructions to quantify the free DNA. The iQuant working solution was prepared by diluting the iQuant dsDNA HS reagent 1:200 in 1× iQuant dsDNA HS Buffer just prior to use. The diluted working solution (190 μL) was added to each well of a black 96-well clear-bottom microplate (Greiner, Kremsmünster, Austria), followed by addition of 10 μL of each dsDNA standard (to obtain the standard curve), or the NP supernatants, in duplicate and mixed thoroughly using pipetting. Microplate was incubated for 2 min in the dark, and fluorescence measurements were obtained using the SpectraMAX Microplate Reader (Molecular Devices, San Jose, CA, USA), with an excitation wavelength of 485 nm and emission at 530 nm.

#### 2.4.3. Nanoparticle Morphology Analysis

The morphology of mcDNA-E7mut-E6mut/RALA/R8M NP was analyzed by transmission electron microscopy (TEM). For sample preparation, the NPs were formulated and subjected to centrifugation at 10,000× *g* for 20 min at 4 °C, followed by a washing step with 1 mL of ultrapure-grade water and a second centrifugation. The resulting pellet was resuspended in 100 µL of ultrapure-grade water and sonicated for 1 min. A 20 µL drop of the NPs suspension was placed on a surface, and a formvar-coated copper grid with carbon (Sigma Aldrich, Lisbon, Portugal) was positioned on the drop for 1 min. Afterward, the carbon grid was left to dry overnight. Staining was performed with 2% (*w*/*v*) uranyl acetate for 1 min and allowed to dry for 10–15 min at room temperature. The TEM imaging was carried out using a Hitachi HT7700 microscope (Hitachi High Technologies, Tokyo, Japan), operated at an accelerating voltage of 80 kV.

#### 2.4.4. Identification of Functional Groups on Nanoparticles

Fourier transform infrared spectroscopy (FTIR) was applied to investigate the functional groups on the surface of each NPs type. For this analysis, 10 μL of each NP formulation (binary and ternary systems) and the individual components (RALA, R8M and mcDNA) were examined using a Nicolet iS10 FTIR spectrophotometer (Thermo Scientific, Waltham, MA, USA) in ATR mode with a Smart iTR diamond ATR accessory. A total of 120 scans were recorded across the spectral range of 4000–800 cm^−1^, with a resolution of 32 cm^−1^.

#### 2.4.5. Conditions of Stability and Decomplexation Assays

Stability assays were performed using mcDNA-E6/RALA and mcDNA-E6/RALA/R8M nanosystems. Following formulation, the NPs were centrifuged, and the resulting pellets were resuspended in 14 μL of either Minimum Essential Medium Alpha Modification (MEM-α) medium (Cytiva-HyClone Laboratories Inc., Logan, UT, USA) or 10% (*v*/*v*) heat-inactivated Fetal Bovine Serum (FBS). Samples were incubated at 37 °C for 0 h and 6 h. The release and potential mcDNA degradation were evaluated by 0.8% agarose gel electrophoresis. To assess decomplexation of the nanosystems, sodium dodecyl sulfate (SDS) was added to each sample previously resuspended in 14 µL of MEM-α or 10% FBS, as described above, after the incubation periods of 0 h and 6 h. Specifically, 1.5 µL of 10% (*w*/*v*) SDS was added to each sample, resulting in a final volume of 15.5 µL and a final SDS concentration of 0.97% (*w*/*v*). Samples were then incubated at room temperature for 10 min. The integrity of mcDNA after decomplexation was subsequently analyzed by agarose gel electrophoresis under the same conditions.

### 2.5. In Vitro Studies

#### 2.5.1. Cell Culture and Growth Conditions

This study used murine immature DCs, JAWS II (ATCC^®^, CRL-11904^TM^). Cells were cultured in MEM-α medium supplemented with 10% (*v*/*v*) heat-inactivated FBS, 5 ng/mL granulocyte-macrophage colony-stimulating factor (GM-CSF) (Shenandoah Biotechnology, Inc., Warminster, PA, USA), and a combination of antibiotics: penicillin (100 μg/mL) and streptomycin (100 μg/mL). Cell growth was maintained at 37 °C in a humidified atmosphere containing 5% CO_2_.

#### 2.5.2. Cell Seeding and Transfections

The JAWS II cells were initially seeded in appropriate culture plates, depending on the assay type to be performed. Seeding was performed at a density of 3 × 10^4^ cells/cm^2^ with complete medium. After 24 h, the culture medium was replaced with medium without supplements to induce nutritional starvation, which may help in enhancing transfection efficiency. Transfections were carried out by adding 4 µg of encapsulated DNA per 12 × 10^4^ cells. Six h post-transfection, the medium without supplements was replaced with complete medium to halt the transfection process, and the cells were maintained in culture according to the specific endpoint of each assay.

#### 2.5.3. Cell Viability Study

The biocompatibility of the delivery systems was evaluated using a modified reduction resazurin-based viability assay [36]. The JAWS II cells were seeded in 96-well plates at a density of 10^4^ cells per well. For transfection, the three mcDNA vectors, either non-encapsulated or encapsulated with RALA or RALA/R8M systems, were added to the cells. The NPs were centrifuged at 10,000× *g* for 20 min at 4 °C, and the pellet was resuspended in medium without supplements before the application into the cells. Cell viability was assessed at 24 and 48 h post-transfection. At each time point, the culture medium was removed and replaced with 200 µL of a fresh medium without supplements containing 2.6 µL of 0.1% (*w*/*v*) resazurin solution. Empty wells containing the resazurin-medium mixture served as blanks. Additionally, non-transfected cells were used as negative control (K−) and ethanol-treated cells were used as positive control (K+). Plates were incubated in the dark for 4 h at 37 °C, after which absorbance was measured at 570 nm and 600 nm using an xMark™ Microplate Absorbance Spectrophotometer (Bio-Rad Laboratories, Algés, Portugal). The percentage of resazurin reduction was calculated using the following formula:

(1) $$\mathrm{Reduction}\;\mathrm{Percentage}\;=\;\frac{\left(\varepsilon OX_{600nm}\times A_{570nm}\_t_{x}\;-\varepsilon OX_{570nm}\times A_{600nm}\_t_{x}\;\right)}{\left(\varepsilon {RED}_{570nm}\times A_{600nm}\_t_{0}\;-\varepsilon RED_{600nm}\;\times A_{570nm}\_t_{0}\right)}$$

where *ε* represents the molar extinction coefficients of Resazurin (for the reduced form [*RED*]: 155,677 at 570 nm and 14,652 at 600 nm; for the oxidized form [*OX*]: 80,586 at 570 nm and 117,216 at 600 nm), *A* is the absorbance at a given wavelength, *t*_0_ is the initial measurement (non-reduced sample/blank), and *tₓ* is the measurement at time *x* (24 or 48 h). Cell viability was expressed as a percentage relative to non-transfected controls.

#### 2.5.4. Reverse Transcription Polymerase Chain Reaction

Reverse transcription polymerase chain reaction (RT-PCR) was used to evaluate the mRNA expression levels of E7mut and E6mut genes encoded by the mcDNA vectors following transfection. For this purpose, JAWS II cells were seeded in 12-well plates at a density of 1.2 × 10^5^ cells per well and transfected with 4 μg of mcDNA (E7mut, E6mut and E7mut-E6mut), encapsulated in either RALA or RALA/R8M NPs. After 24 h of transfection, the culture medium was removed, and cells were lysed directly in the culture plate by adding 350 μL of TripleXtractor reagent (GRISP, Porto, Portugal) to each well. Cell lysis was achieved by repeatedly pipetting the lysate up and down, followed by incubation for 5 min at room temperature. The resulting lysates were then transferred to microcentrifuge tubes, and total RNA was extracted using a chloroform-based phase separation method, as described in a previous study from our research group [31]. RNA integrity was assessed by 1% (*w*/*v*) agarose gel electrophoresis, and RNA quantification was performed using a NanoPhotometer^®^. Complementary DNA (cDNA) was synthesized using 1 µg of total RNA and the Xpert cDNA Synthesis Kit (GRISP, Porto, Portugal), according to the manufacturer’s instructions. Each PCR reaction contained 4.45 μL of sterile ultra-grade pure water, 0.16 μM of forward primer (FW), 0.16 μM of reverse primer (RV), 6.25 μL of NZYTaq II 2× Green Master Mix (NZYTech, Lisbon, Portugal) and 1 μL of cDNA. The primers used for E7mut detection were FW (5′-ATG CCT GGA GAT ACA CCT ACA TT-3′) and RV (5′-AAC CGA AGC GTA GAG TCA CA-3′), while for E6 mut were FW (5′-GCG ACC CAG AAA GTT ACC AC-3′) and RV (5′-CAC AGT GGC TTT TGA CGG TTA-3′). After gentle homogenization, the reaction mixtures were processed in a T100™ Thermal Cycler (Bio-Rad Laboratories Inc., Hercules, CA, USA) under the following conditions: initial denaturation at 95 °C for 5 min; 25 cycles of denaturation at 95 °C for 30 s, annealing at 56 °C for 30 s, and extension at 72 °C for 60 s; followed by a final extension step of 72 °C for 10 min. PCR products were analyzed by 1% (*w*/*v*) agarose gel electrophoresis.

#### 2.5.5. Quantitative Reverse Transcription Polymerase Chain Reaction (RT-qPCR)

The levels of E7mut and E6mut transcripts in cells transfected with the RALA-based systems were analyzed by RT-qPCR. Each reaction mixture contained 10 μL of Xpert Fast SYBR Mastermix (Grisp, Porto, Portugal), 0.16 μM of FW primer, 0.16 μM of RV primer, 1 μL of cDNA, and nuclease-free water to a final volume of 20 μL. The housekeeping gene GAPDH was used for normalization of expression levels. The primer sequences for GAPDH were FW (5′-ATG GGG AAG GTG AAG GTC G-3′) and RV (5′-GGG GTC ATT GAT GGC AAC AAT A-3′). Reactions were performed in a CFX Connect™ Real-Time PCR system (Bio-Rad Laboratories, Hercules, CA, USA) under the following thermal profile: 95 °C for 5 min, followed by 40 cycles at 95 °C for 30 s, 56 °C for 30 s, and 72 °C for 60 s. Relative expression levels of E7 mut and E6mut were calculated using the comparative threshold cycle (Ct) method (^−^ΔΔCt), normalized to the GAPDH housekeeping gene.

For the evaluation of cytokine expression, specifically TNF-α and IL-6, JAWS II cells were transfected following the same protocol described above using all mcDNA/RALA and mcDNA/RALA/R8M NP formulations. Cells were lysed and harvested 12 h post-transfection. Cytokine expression was measured at the mRNA transcript levels by RT-qPCR using the same Xpert Fast SYBR Mastermix and normalization to GAPDH. Primer sequences were as follows: TNF-α FW (5′-CTA CTC CCA GGT TCT CTT CA-3′) and RV (5′-TGA CTC CAA AGT AGA CCT GC-3′); IL-6 FW (5′-CAA GAA AGA CAA AGC CAG AG-3′) and RV (5′-TTG GAT GGT CTT GGT CCT TA-3′). The same thermal cycling profile was applied, except for the annealing temperature, which was adjusted to 55 °C for cytokine amplification.

#### 2.5.6. E7 Protein Analysis and Mannose-Receptor Competitive Inhibition Assay

JAWS II cells were seeded in 6-well plates at a density of 3 × 10^5^ cells per well. The mcDNA-E7mut/RALA/R8M nanoparticles were transfected at 10 µg per well. To evaluate mannose receptor-dependent uptake, a competitive inhibition assay was performed by pre-incubating cells with culture medium containing D-mannose (20 mg/mL) 30 min prior to transfection. After a 48 h incubation period, cells were collected and lysed with a buffer containing 25 mM Tris-HCl (pH 7.4), 2.5 mM EDTA, 1% Triton X-100, 2.5 mM EGTA, 2.5 mM phenylmethylsulfonyl fluoride, and an EDTA-free protease inhibitor cocktail (Roche, Basel, Switzerland). Lysates were incubated on ice for 10 min, clarified by centrifugation at 10,000× *g* for 1 min at 4 °C, and total protein content was determined using the Pierce BCA Protein Assay Kit (Thermo Scientific, Waltham, MA, USA).

The E7 protein levels were quantified using a commercial HPV16 E7 SimpleStep ELISA^®^ kit (Abcam, Amsterdam, The Netherlands) according to the manufacturer’s instructions. Lysates were normalized to 0.1 µg/µL total protein, and 50 µL of each sample was added to the ELISA plate along with 50 µL of the antibody cocktail. Plates were incubated for 1 h at room temperature with 400 rpm agitation, washed three times, and 100 µL of TMB substrate was added. After 15 min in the dark, the reaction was stopped with 100 µL stop solution, and absorbance was measured at 450 nm using an xMark™ Microplate Spectrophotometer (Bio-Rad, Hercules, CA, USA). Protein concentration was calculated from the standard curve and corrected for dilution factors.

### 2.6. Data Processing and Statistical Analysis

Data were processed and analyzed using GraphPad Prism (version 8.01). The results are presented as the mean ± standard deviation (SD) of three independent experiments per assay. The data obtained from the NPs optimizations (hydrodynamic diameter, PDI, zeta potential and CE) were analyzed using two-way Analysis of Variance (ANOVA) followed by Tukey’s multiple comparison test. The results of DLS and CE of the NPs, as well as the RT-qPCR results and E7 protein levels by ELISA, were analyzed using one-way ANOVA, followed by Tukey’s multiple comparisons test. For the viability study, statistical analysis was performed using two-way ANOVA followed by Sidak’s multiple comparisons test. A *p*-value of less than 0.05 was considered statistically significant.

## 3. Results

### 3.1. Optimization of RALA Concentration for mcDNA Complexation

The optimization of RALA/mcDNA formulations was performed by testing increasing concentrations of RALA (29, 58, 72.5, 81.2, 87, and 116 µg/mL), complexed with 2 µg of mcDNA-E7mut, in the absence or presence of R8M (fixed concentration at 58 µg/mL).

**Figure 1:**
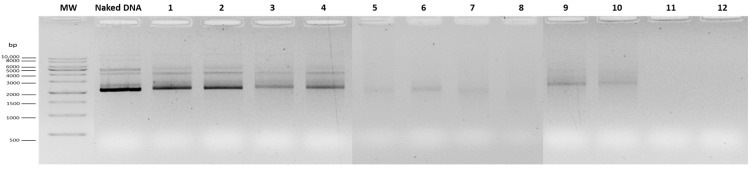
Agarose gel electrophoresis of supernatants from mcDNA-E7mut/RALA and mcDNA-E7mut/RALA/R8M NPs. Lanes 1–8 correspond to the respective formulations listed in Table 1. MW: molecular weight marker GRS ladder 1 kb (Grisp^®^). Naked DNA is non-encapsulated mcDNA-E7mut sample for comparison.

At 29 µg/mL, NPs exhibited a hydrodynamic diameter below 200 nm with slightly negative zeta potential (−5.83 ± 0.18 mV) but presented high PDI (0.60 ± 0.10) (Table 1) and poor DNA complexation, as evidenced by a substantial amount of uncomplexed DNA as evidenced on agarose gel (Figure 1). The addition of R8M under this condition further increased sample heterogeneity, leading to excessive PDI and scattering interference, which rendered DLS data acquisition unfeasible.

At 58 µg/mL, the DNA complexation was improved, although some amount of free DNA was still detectable in the agarose gel (Figure 1). Despite numerical differences, the particle hydrodynamic diameter, PDI and surface charge values were not statistically different from the previous tested concentration. Intermediate concentrations (72.5 and 81.2 µg/mL) were evaluated. At 72.5 µg/mL, NPs showed the most favorable characteristics, with significantly smaller hydrodynamic diameter compared to all tested concentrations (*p* < 0.01; Table 1, group letters d vs. a–c), suitable surface charge (−10.56 ± 3.47 mV), and high DNA complexation (Figure 1). No significant difference was observed with R8M at this concentration. At 81.2 µg/mL, the presence of R8M significantly increased the particle size compared to both the non-mannosylated formulation at the same concentration and the 72.5 µg/mL R8M formulation (*p* < 0.05–0.001; Table 1). At 87 µg/mL, NPs’ size remained low (125–139 nm, depending on R8M), PDI was acceptable, and DNA complexation was nearly complete. At 116 µg/mL, NPs aggregated, preventing reliable DLS measurement, despite full DNA complexation (Figure 1, Table 1). Overall, 72.5 µg/mL RALA was identified as the optimal concentration, ensuring efficient DNA complexation, suitable physicochemical properties, and consistent inclusion of R8M without compromising NP properties.

### 3.2. Physicochemical Characterization of Optimized RALA-Based Nanoparticles

#### 3.2.1. Hydrodynamic Diameter, Polydispersity Index, Zeta Potential, and Complexation Efficiency

The NPs were formulated using 2 µg of each mcDNA vector (E7mut, E6mut, and E7mut-E6mut), complexed with RALA at 72.5 µg/mL, the concentration previously identified as optimal, considering the most favorable physicochemical properties (hydrodynamic diameter, particle homogeneity, surface charge and complexation efficiency). The R8M was incorporated at 58 µg/mL in selected formulations to evaluate the impact of mannose functionalization. The physicochemical properties, including hydrodynamic diameter, PDI, zeta potential, and DNA CE, were assessed by DLS and fluorescence quantification, respectively (Table 2).

Across all formulations, NPs presented favorable characteristics: hydrodynamic diameter below 150 nm, PDI values under 0.3, and negative surface charges. CE values exceeded 97% in all groups. Statistical analysis (one-way ANOVA followed by Tukey’s post-test) revealed no significant differences (*p* > 0.05) among the evaluated parameters, regardless of vector type or R8M inclusion. These results were expected, as the only variable in the formulations was the DNA insert; all vectors were similar in size and composition. Moreover, no significant differences were observed between binary (mcDNA/RALA) and ternary (mcDNA/RALA/R8M) systems, corroborating previous findings from the optimization screening.

#### 3.2.2. Morphological Evaluation by Transmission Electron Microscopy

**Figure 2:**
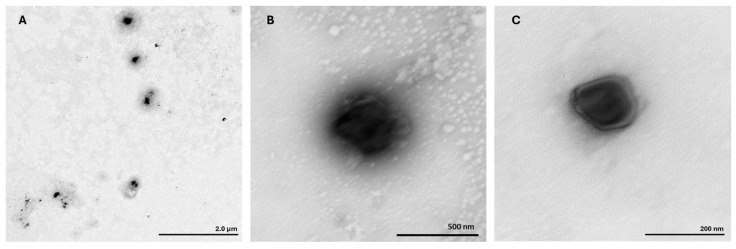
Transmission electron microscopy images of mcDNA/RALA/R8M NPs (2 µg), formulated with RALA at 72.5 µg/mL and R8M at 58 µg/mL concentration. (**A**) mcDNA-E7mut/RALA/R8M NP; (**B**) mcDNA-E6mut/RALA/R8M NP; and (**C**) mcDNA-E7mut-E6mut/RALA/R8M NP.

The TEM analysis was performed to assess the morphology of ternary mcDNA/RALA/R8M NPs (Figure 2).

The images revealed well-dispersed, spherical particles ranging from 47 nm to 190 nm, with the majority measuring close to 80 nm. These results were in agreement with the hydrodynamic diameters previously measured by DLS. No signs of aggregation were observed, confirming the structural stability and monodispersity of NPs formulations.

#### 3.2.3. FTIR Analysis

**Figure 3:**
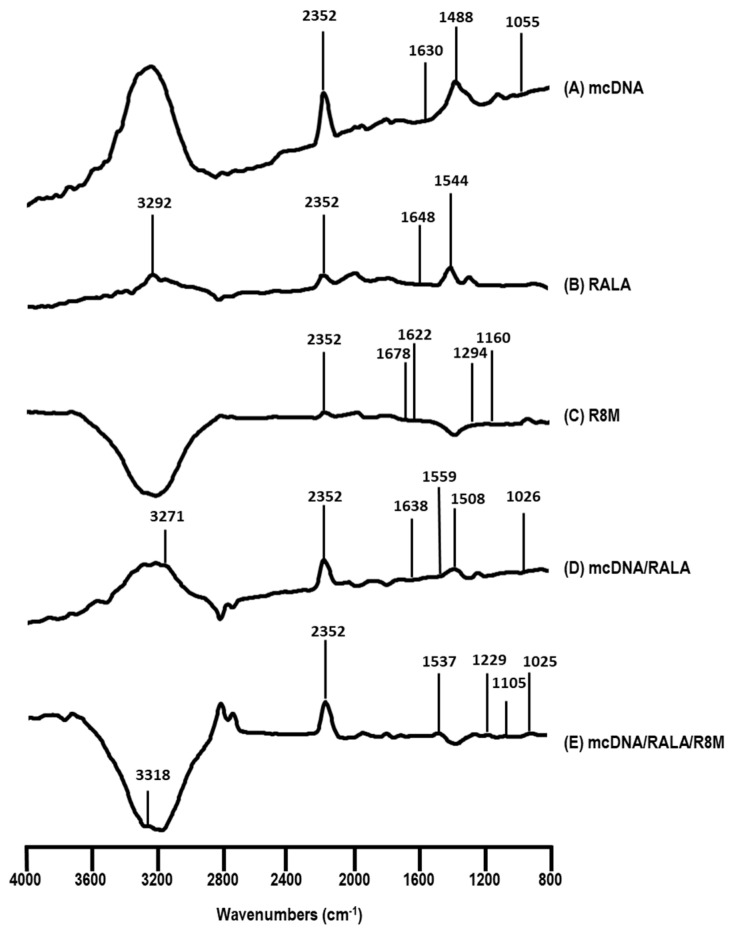
FTIR spectra (absorbance versus wavenumber) of mcDNA (**A**), RALA (**B**), R8M (**C**), mcDNA/RALA NP (**D**), and mcDNA/RALA/R8M NP (**E**).

The FTIR technique was conducted to confirm the presence of all components and investigate interactions within the NP formulations. The corresponding spectra are shown in Figure 3.

The FTIR spectrum of mcDNA (A) shows characteristic peaks at 1630, 1488, and 1055 cm^−1^, corresponding to the vibration of nitrogenous bases and the ribose, which align with previously published data [37,38]. For RALA spectrum (B), peaks at 3292 cm^−1^ and 1544 cm^−1^ are attributed to C-H stretching and amine/carboxyl group vibrations, respectively [38,39]. The R8M spectrum (C) displays peaks at 1294 cm^−1^ and 1160 cm^−1^, associated with C=O and N-C stretching, with evidence of mannose presence [32,40,41]. In the mcDNA/RALA in spectrum (D), peaks at 3271 cm^−1^ and 1559 cm^−1^ confirm the presence of RALA, while peaks at 1638, 1508, and 1026 cm^−1^ correspond to mcDNA, indicating successful complex formation. In the mcDNA/RALA/R8M spectrum (E), peaks at 3318 and 1537 cm^−1^ indicate the presence of RALA, while peaks at 1229 and 1105 cm^−1^ correspond to R8M, and a peak at 1025 cm^−1^ confirms the presence of mcDNA. Shifts in some peaks suggest interactions between the components, likely due to the complexation process. The absence of certain characteristic peaks, such as the ribose peak, suggests that the interactions involve the sugar-phosphate backbone of the mcDNA molecule. Additionally, the peak at 2352 cm^−1^, observed across all spectra, likely results from incomplete CO_2_ purging of the spectrometer, as reported in other studies [38,39].

#### 3.2.4. Stability and Decomplexation Assays

**Figure 4:**
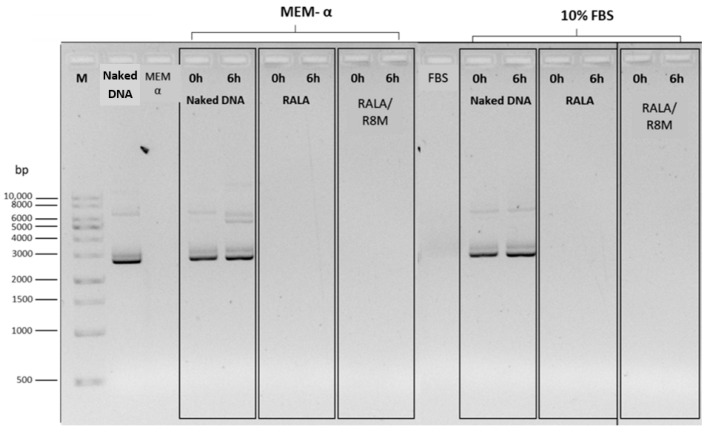
Evaluation of the stability of mcDNA-E6mut/RALA and mcDNA-E6mut/RALA/R8M NPs by agarose gel electrophoresis. Incubations were performed at 37 °C for 0 and 6 h in MEM-α medium or 10% FBS. Control lanes only include MEM-α medium and 10% FBS. M: Molecular weight marker. DNA and Naked DNA refer to the non-encapsulated mcDNA-E6mut vector.

To evaluate NP stability under physiological conditions, formulations were incubated in MEM-α without supplements and in 10% FBS for 6 h at 37 °C. Agarose gel electrophoresis (Figure 4) revealed no detectable mcDNA release at either time point (0 h and 6 h), indicating that the DNA remained stably complexed into the NPs in both incubation conditions.

**Figure 5:**
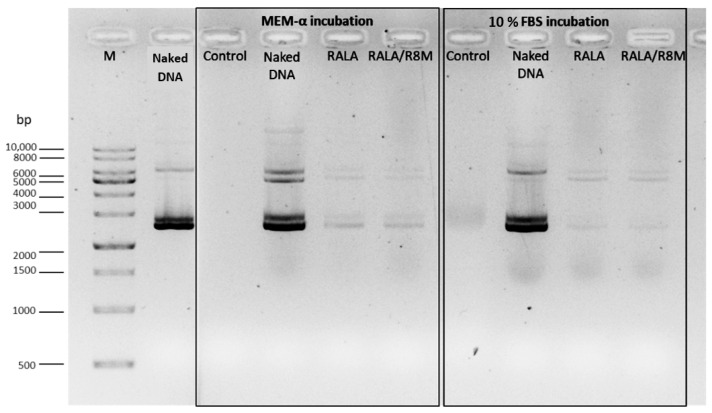
Analysis of mcDNA-E6mut/RALA NPs decomplexation, with and without R8M, by agarose gel electrophoresis. The NPs, along with naked DNA, were incubated for 6 h at 37 °C in MEM-α medium or 10% FBS, followed by decomplexation with 1.5 µL of 10% SDS. Controls lanes include MEM-α medium and 10% FBS. M: Molecular weight marker. Naked DNA refers to the non-encapsulated mcDNA-E6mut vector.

Following incubation, the NPs were treated with 10% SDS to promote decomplexation, and samples were subsequently analyzed by agarose gel electrophoresis (Figure 5).

Faint mcDNA bands were observed, confirming that the DNA remained intact after incubation and could be released from the delivery systems. These results indicate that the optimized RALA-based NPs successfully protected and preserved mcDNA integrity under cell culture conditions and simulation of physiological conditions.

### 3.3. Cytotoxicity Evaluation in Dendritic Cells

**Figure 6:**
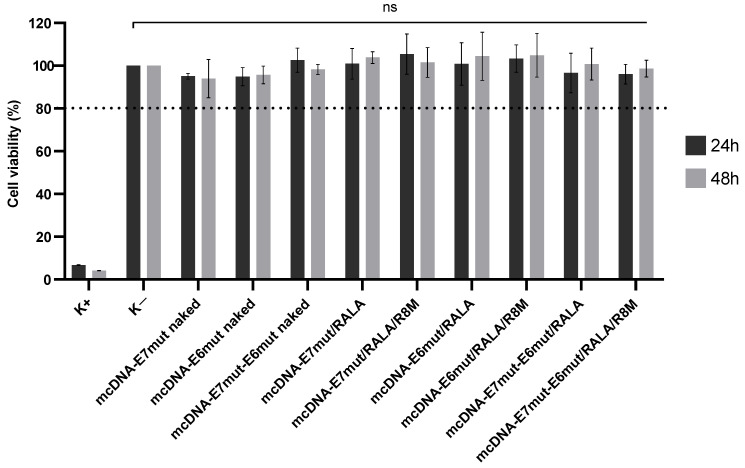
Viability of JAWS II cells transfected with naked mcDNA, mcDNA/RALA, or mcDNA/RALA/R8M NPs encapsulating mcDNA-E7mut, mcDNA-E6mut, or mcDNA-E7mut-E6mut. Cell viability was evaluated using the resazurin reduction assay at 24 and 48 h post-transfection. Data are presented as mean ± SD of the percentage of viable cells relative to the non-transfected control (K−). A positive control (K+) was treated with ethanol to induce cell death. No statistically significant (ns) differences were observed between the transfected groups and K−, whereas K+ showed a significant reduction in cell viability (*p* < 0.001). Dash line evidencing the cell viability above 80%.

The biocompatibility of RALA-based delivery systems was evaluated in JAWS II DCs by assessing cell viability at 24 and 48 h post-transfection. Both binary and ternary formulations, encapsulating each of the three mcDNA vectors, were tested. Resazurin assay results (Figure 6) showed that all treatment groups maintained cell viabilities above 80% (as represented by dash line), with no statistically significant differences compared to non-transfected controls. These findings demonstrate that the formulations are non-cytotoxic and suitable for further cellular applications.

### 3.4. Evaluation of Relative Gene Expression by RT-PCR and RT-qPCR

**Figure 7:**
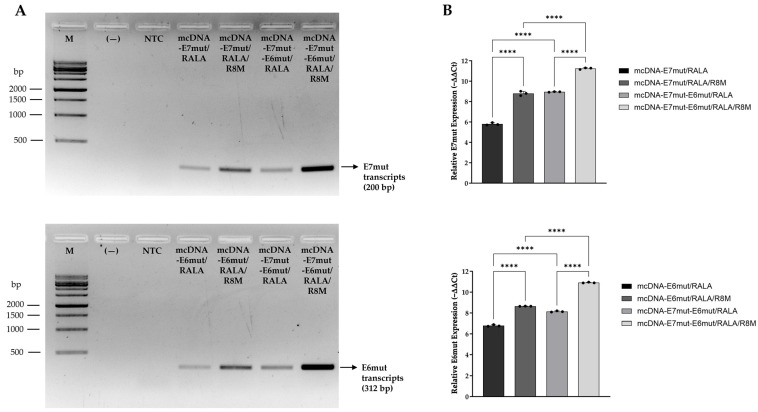
Evaluation of E7mut and E6mut transcript levels in JAWS II cells 24 h after transfection with mcDNA-E7mut, mcDNA-E6mut and mcDNA-E7mut-E6mut vectors encapsulated with RALA or RALA/R8M. (**A**) Detection of E7mut and E6mut transcripts by agarose gel electrophoresis of RT-PCR products. M: Molecular weight marker; (—): Negative control; NTC: Non-transfected cells. (**B**) Quantitative analysis of E7mut and E6mut relative expression by RT-qPCR. Non-transfected cells were used as control. Data are presented as mean ± SD from three independent experiments, with individual dots shown. Expression levels were normalized to the housekeeping gene GAPDH. Statistical significance: **** *p* ≤ 0.0001.

To confirm the transcription of the target genes, E7mut and E6mut transcripts were evaluated in JAWS II cells 24 h after transfection with the RALA-based nanosystems formulated with different mcDNA vectors. Transcript detection was initially performed by RT-PCR and subsequently quantified by RT-qPCR, as shown in Figure 7.

As shown in Figure 7A, RT-PCR analysis confirmed the expression of E7mut and E6mut transcripts in JAWS II cells transfected with RALA or RALA-R8M nanosystems, delivering either monogenic or multigenic mcDNA vectors. No amplification was observed in non-transfected cells, confirming the absence of basal expression of E7mut and E6mut in this cell line. For E7mut, a specific amplification band of approximately 200 bp was detected in all transfected groups. Among them, formulations containing RALA/R8M clearly exhibited more intense bands compared to those containing RALA alone. Additionally, the multigenic mcDNA-E7mut-E6mut vector yielded stronger E7mut amplification than the monogenic mcDNA-E7mut vector. A similar expression profile was observed for the E6mut transcript, with a specific band of approximately 312 bp detected exclusively in transfected conditions. A more pronounced E6mut amplification was observed with RALA/R8M formulations compared to those containing RALA alone. Likewise, the multigenic mcDNA-E7mut-E6mut vector resulted in higher E6mut transcript levels than the single-gene mcDNA-E6mut vector. To validate these findings, RT-qPCR was performed to quantify the relative expression levels of E7mut and E6mut (Figure 7B). When normalized to the non-transfected control, all transfected groups showed increased expression of both genes. Notably, mannosylated nanosystems (RALA-R8M) induced significantly higher expression of both genes when compared to non-mannosylated systems (RALA-only). Furthermore, multigenic vector consistently resulted in greater transcript levels than the corresponding single-gene vectors. Together, these results confirm the successful transcription of both E7mut and E6mut following transfection with the tested nanosystems, with enhanced outcomes observed for mannosylated formulations and the multigenic mcDNA-E7mut-E6mut vector.

### 3.5. Evaluation of Pro-Inflammatory Cytokines Gene Expression by RT-qPCR

**Figure 8:**
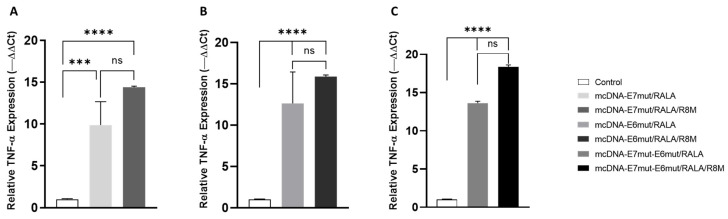
Quantitative analysis of TNF-α transcript expression by RT-qPCR in JAWS II cells 12 h after transfection with (**A**) mcDNA-E7mut, (**B**) mcDNA-E6mut, or (**C**) mcDNA-E7mut-E6mut vectors encapsulated with RALA or RALA/R8M. Data are presented as mean ± SD from three independent experiments. Expression levels were normalized to the housekeeping gene GAPDH. Non-transfected cells were used as the control and showed statistically significant differences (*** *p* < 0.0005; **** *p* < 0.0001; ns: Not Significant) compared with all transfected groups, as determined by one-way ANOVA followed by Tukey’s multiple comparison test.

**Figure 9:**
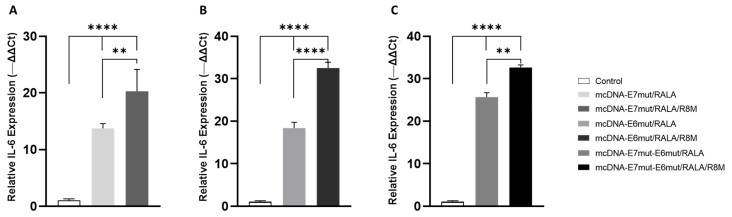
Quantitative analysis of IL-6 transcript expression by RT-qPCR in JAWS II cells 12 h after transfection with (**A**) mcDNA-E7mut, (**B**) mcDNA-E6mut, or (**C**) mcDNA-E7mut-E6mut vectors encapsulated with RALA or RALA/R8M. Data are presented as mean ± SD from three independent experiments. Expression levels were normalized to the housekeeping gene GAPDH. Non-transfected cells were used as the control and showed statistically significant differences (**** *p* < 0.0001) compared with all transfected groups, as determined by one-way ANOVA followed by Tukey’s multiple comparison test. Statistical significance: ** *p* ≤ 0.005.

To investigate whether mcDNA delivery by the RALA-based nanosystems could modulate early inflammatory signaling in dendritic cells, the expression levels of TNF-α and IL-6 were assessed 12 h after transfection. Cytokine quantification was performed by RT-qPCR, using GAPDH as the reference gene, as shown in Figure 8 and Figure 9.

For TNF-α, all transfected groups exhibited a significant increase in mRNA levels compared to non-transfected JAWS II cells, which displayed only basal expression of this cytokine (Figure 8). This confirms that both RALA and RALA/R8M nanosystems are capable of inducing an early pro-inflammatory response following mcDNA delivery. Although the results show a trend toward higher expression of this cytokine with R8M-functionalized systems, this difference is not statistically significant when compared with non-mannosylated systems.

On the other hand, the expression profile of IL-6 revealed a distinct pattern (Figure 9). As observed for TNF-α, all formulations induced a significant upregulation of IL-6 relative to the non-transfected control, confirming successful activation of dendritic-like cells by the tested nanosystems. Notably, mannosylated formulations (RALA/R8M) generated significantly higher IL-6 transcript levels compared to their non-mannosylated counterparts. These findings indicate that the incorporation of the R8-mannose peptide may enhance early IL-6 signaling, potentially reflecting improved uptake or intracellular processing through mannose receptor–mediated pathways.

Together, these results demonstrate that all tested nanosystems are capable of stimulating early cytokine responses in JAWS II cells, with the influence of R8M functionalization more evident and significant in IL-6 cytokine.

### 3.6. Evaluation of E7 Protein Levels and Mannose-Receptor Mediated Uptake

**Figure 10:**
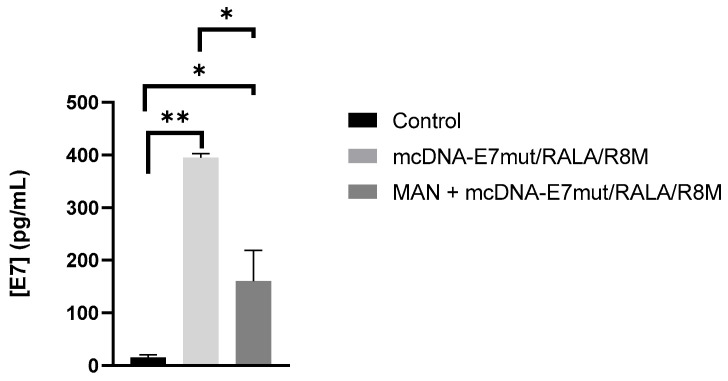
E7 protein expression in JAWS II cells transfected with mcDNA-E7mut/RALA/R8M nanoparticles. E7 levels were measured 48 h post-transfection by sandwich ELISA. Mannose receptor-mediated uptake was assessed by pre-incubating cells with 20 mg/mL D-mannose 30 min before transfection. Data are mean ± SD of two independent experiments. Statistical significance: * *p* ≤ 0.05; ** *p* ≤ 0.003.

To further explore and confirm the role of mannose ligands in the performance of the RALA/R8M nanosystems, a sandwich ELISA assay was performed using only the fully functionalized mcDNA-E7mut/RALA/R8M formulation, based on the availability of the E7 protein detection kit. This analysis aimed to (i) confirm E7 protein expression following transfection with the mannosylated nanosystem and (ii) assess the potential contribution of mannose receptor–mediated uptake, after competitively saturating mannose receptors. The results in Figure 10 showed that cells transfected with the mannosylated nanosystem exhibited increased levels of E7 protein expression compared to non-transfected controls. Importantly, pre-incubation with free mannose resulted in a significant reduction in E7 protein levels, suggesting that cellular uptake and subsequent antigen expression are at least partially dependent on mannose receptor engagement. The observed decrease in protein expression upon receptor saturation is consistent with a mannose receptor–mediated contribution to nanosystem internalization.

## 4. Discussion

In the present study, we developed and evaluated RALA-based NPs, functionalized with R8M, for the delivery of mcDNA vectors encoding oncogenes from HPV-16, aiming at their application in DNA vaccines for cervical cancer immunotherapy. The designed delivery systems were extensively characterized in terms of physicochemical properties, stability, biocompatibility, and gene expression performance in APCs, particularly DCs.

The obtained hydrodynamic diameters in the range of 87 and 123 nm, combined with a narrow size distribution, as indicated by the PDI values (<0.3), represent critical factors for enhanced cellular uptake and reproducible cellular transfection, respectively [14,42,43,44,45,46,47,48]. Similarly, results obtained after the inclusion of R8-mannose conjugate did not significantly alter the physicochemical properties of the NPs, but rather added potential for targeted delivery. This result aligns with previous studies indicating that mannose functionalization typically does not significantly influence NPs’ size [32,49].

Despite the cationic nature of RALA, NPs formulated with mcDNA vectors displayed a negative surface charge, even when R8M was added, which also possesses a predominant cationic net charge. This result may be attributed to the high mcDNA CE (>97%), which likely leaves negatively charged phosphate groups exposed on the NPs surface. Additionally, the concentration of RALA (72.5 µg/mL), while sufficient for complexation, may not fully neutralize the net charge. In formulations containing R8M, the R8 peptide might not have significantly contributed to surface charge modulation, possibly due to limited interaction with RALA, which primarily associates with mcDNA.

Although a positive zeta potential is generally associated with enhanced cellular uptake via electrostatic attraction to negatively charged membranes, RALA’s internalization mechanism is not solely dependent on electrostatic interactions. Due to its amphipathic nature, RALA enables membrane destabilization and pore formation, allowing direct delivery of mcDNA into the cytoplasm. Moreover, RALA promotes clathrin-mediated endocytosis and facilitates endosomal escape through interactions with lipid membranes, key steps to prevent degradation and enhance gene delivery efficiency [50]. Meanwhile, the R8 peptide relies primarily on electrostatic interactions for membrane association and entry [50]. For mannose-functionalized NPs, internalization is more likely driven by specific interactions with mannose receptors, particularly CTLD4 domains, which mediate endocytosis and antigen presentation [29].

Altogether, while surface charge and targeting ligands influence NP-cell interactions, other factors such as NP size, shape, and structural organization also play a decisive role in cellular uptake and transfection efficiency [50]. The high CE underscores the stable complex formation between mcDNA and the peptides (RALA and R8M). Such efficiency can ensure that most of the genetic material is protected from degradation by extracellular nucleases and is released only after cellular internalization [51].

Additionally, the spherical and homogeneous morphology observed by TEM is in accordance with desirable features for DNA nanovaccines, ensuring enhanced internalization and transfection efficiency [14,42,43,44,45,46,47,48]. Furthermore, FTIR analysis confirmed the inclusion of each component in the respective formulation, revealing the successful formation of mcDNA/RALA and mcDNA/RALA/R8M conjugates. All the findings are consistent with prior reports on RALA-based delivery systems [26,46,52].

After confirming the physicochemical characteristics of the NPs, their stability was further evaluated through incubation and decomplexation assays designed to mimic the conditions used during in vitro cellular transfection. Agarose gel electrophoresis demonstrated that the nanoparticles effectively protected the mcDNA, preventing its premature decomplexation and degradation for up to 6 h under cell culture conditions. Most of the mcDNA remained associated with the nanosystems during this incubation period, as evidenced by the absence or minimal presence of free DNA bands. Importantly, even after forced decomplexation with SDS, the released mcDNA maintained its structural integrity, indicating that the nanosystems were capable of preserving the DNA during incubation and releasing it without inducing detectable damage. This incubation period was intentionally selected to reflect the duration of the transfection step, because after this time the nanoparticle-containing medium was removed, cells were washed, and fresh FBS-supplemented medium was added, making it unlikely that a relevant fraction of non-internalized nanosystems would remain in the extracellular environment. Overall, these results indicate that the nanosystems could be able to maintain mcDNA stability throughout the extracellular phase of transfection, minimizing premature decomplexation or degradation and thereby supporting further in vitro transfection assays [14].

The results of cell viability studies confirmed the biocompatibility of the tested formulations, considering that cell viability above 80% is generally regarded as non-cytotoxic [53]. This finding aligns with previous studies reporting high cell viability after transfection with RALA-based NPs in various cell lines, such as HEK-293 (human embryonic kidney cells), DC 2.4 (murine DCs) [46], and fibroblasts (connective tissue cells) [52]. These results further confirm the safety of CCP-based systems, namely containing RALA and R8, and their potential for use in a range of in vitro assays involving cell transfection.

Then, the expression of both E7mut and E6mut transcripts was evaluated in JAWS II cells following transfection with RALA-based nanosystems carrying monogenic or multigenic mcDNA vectors. Clear differences in transcript levels were observed between tested formulations. Non-transfected cells showed no detectable expression of either E7mut or E6mut, confirming the specificity of the primers and the absence of endogenous expression in this DC line. In contrast, all transfected conditions showed successful transcription of the target genes, indicating that the mcDNA vectors were effectively delivered and processed by the cells. Notably, mannose-functionalized nanosystems (RALA-R8M) induced significantly higher transcript levels for both E7mut and E6mut compared to non-functionalized RALA nanosystems. This trend was evident in both qualitative (RT-PCR) and quantitative (RT-qPCR) analysis. All transfection conditions resulted in statistically significant increases in gene expression compared with non-transfected controls, with the highest levels observed in the presence of mannose ligands.

However, RALA-only formulations also promoted detectable and relevant expression of E7mut and E6mut transcripts, demonstrating that RALA can function as an effective gene delivery system even without additional modifications. Although expression levels were significantly lower than those obtained with RALA-R8M, these results confirm the intrinsic ability of RALA to mediate gene delivery to APCs, although with reduced efficiency. This observation is consistent with previous reports. For example, Cole and collaborators demonstrated gene expression following transfection with pDNA/RALA complexes in HEK-293 and DC 2.4 cells, with higher transfection efficiency and gene expression in HEK-293 cells, which corroborates the fact that APCs, such as DCs, are inherently more challenging to transfect [46]. Similarly, McCarthy and co-workers showed that pDNA/RALA complexes achieved transfection efficacy both in vitro and in vivo [47]. These studies suggest that the performance of RALA alone can be efficient depending on the application, concentration, and route of administration. Neves and colleagues further emphasized the relevance of the amine-to-phosphate (N/P) ratio in RALA formulations, showing that it significantly affects nanoparticle properties and transfection efficiency [52]. While our study focused on optimizing formulation concentrations, further fine-tuning of the N/P ratio could contribute to additional improvements in gene expression outcomes.

To specifically confirm mannose receptor-mediated uptake, mannose-functionalized RALA/R8M nanoparticles were evaluated using a competitive inhibition assay with free D-mannose. Cells transfected with the functionalized system showed significantly higher E7 protein expression compared to non-transfected controls. Pre-incubation with D-mannose markedly reduced protein levels, indicating that uptake was at least partially mediated by mannose receptors. These results highlight the contribution of mannose ligands to enhanced nanoparticle internalization and targeted antigen delivery to APCs, reinforcing the potential of ligand-functionalized CPP-based systems for DNA vector delivery [28,29,54].

The superior performance of RALA-R8M formulations in this study may stem from the synergistic effects of improved mcDNA protection by the R8 peptide and enhanced receptor-mediated uptake via mannose ligands. These findings underscore the benefit of rational nanosystem design for improving transfection efficiency in hard-to-transfect immune cells like APCs. Additionally, the multigenic mcDNA-E7mut-E6mut vector consistently promoted higher transcript levels than the corresponding single-gene vectors, supporting its application in co-expression strategies and future therapeutic development. In summary, while RALA alone is capable of mediating gene delivery and expression in dendritic cells, the functionalization of these NPs with R8 and mannose was for the first time explored in this work and confirmed a significant improvement of the NPs performance, particularly in APC-targeted applications. These findings align with those of Serra et al., who demonstrated that PEI/R8M systems achieved higher gene expression in RAW 264.7 macrophages than PEI alone, with preferential transfection of APCs over fibroblasts [30]. Similarly, Eusébio and collaborators showed enhanced gene and protein expression in JAWS II cells using mannose-functionalized delivery systems [31]. Costa and collaborators also demonstrated that mannose-functionalized solid lipid NPs achieved superior uptake by alveolar macrophages, which was significantly reduced in the presence of free mannose, confirming receptor-specific internalization [32]. Moreover, another study using mannose-functionalized antigen nanoparticles (MAN-OVA/PEI) reported improved dendritic cell uptake, cytosolic release, MHC I presentation, and cytokine secretion [54]. Together, these studies reinforce the effectiveness of mannose ligands in targeting nanocarriers to APCs via receptor-mediated mechanisms, supporting the benefits observed with RALA-R8M systems in our study.

Building on the observed gene expression profiles, we next assessed how transfection with RALA and RALA-R8M nanosystems influences the induction of pro-inflammatory cytokines in JAWS II cells, providing insight into the potential immunostimulatory effects of these formulations. Dendritic cells play a pivotal role in initiating immune responses by capturing, processing, and presenting antigens to T cells. Upon activation, DCs undergo maturation characterized by upregulation of co-stimulatory molecules, chemokine receptors, and secretion of pro-inflammatory cytokines such as TNF-α and IL-6 [55,56]. This cytokine production not only reflects DC activation but also is essential for promoting subsequent T-cell proliferation and adaptive immunity [57,58].

In this study, the transcriptional levels of TNF-α and IL-6 were evaluated in JAWS II cells 12 h post-transfection with RALA or RALA/R8M nanoparticles carrying different mcDNA vectors. Our results indicate that TNF-α transcript levels were significantly elevated in all transfected groups compared to non-transfected cells, which exhibited only basal expression. In general, the mannosylated systems increase the cytokine transcripts expression when compared with non-mannosylates systems, being a significant effect only noted in the case of IL-6 cytokine.

These findings can be interpreted in light of the known regulatory mechanisms of cytokine production in DCs. TNF-α is rapidly induced upon activation and is often one of the earliest cytokines expressed, contributing to autocrine and paracrine signaling that reinforces DC maturation [55,56]. The absence of significant differences between mannosylated and non-mannosylated systems for TNF-α might reflect a ceiling effect at the transcriptional level after 12 h, whereby maximal induction is reached irrespective of enhanced uptake through mannose receptor-mediated endocytosis. Conversely, IL-6 expression appears more sensitive to enhanced nanoparticle internalization, consistent with studies showing that increased antigen uptake or processing can preferentially upregulate certain cytokines [56,57]. The higher IL-6 levels observed with mannosylated NPs suggest that targeted delivery to endocytic pathways enriched in mannose receptors may amplify specific cytokine transcription, in line with their role in promoting DC maturation and T-helper cell priming [57,58]. In addition, the E7 expression level assessed by ELISA kit also proved the role and importance of the mannose ligands in the NPs to enhance cellular mannose-receptor-mediated uptake and subsequent antigen expression.

Overall, these results confirm that both RALA and RALA/R8M nanosystems effectively activate immature DCs, as indicated by the production of key pro-inflammatory cytokines. While TNF-α induction seems robust across all formulations, the increased IL-6 levels with mannosylated systems highlight the potential advantage of ligand-functionalized CPP-based nanoparticles for fine-tuning immune responses. These insights provide a strong rationale for future in vivo studies to evaluate the immunogenicity and efficacy of mcDNA vaccines delivered via these CPP-based nanosystems.

In addition to their potential for APC targeting and maturing, mannose ligands may offer advantages depending on the administration route. For example, mucosal vaccination, including intranasal or pulmonary delivery, provides direct access to APC-rich tissues such as macrophages, dendritic cells, and Langerhans cells. In this context, mannose ligands could facilitate rapid recognition and uptake via C-type lectin receptors, potentially reducing nanoparticle exposure to serum components before internalization [29].

## 5. Conclusions

In conclusion, the RALA-based NPs developed in this study, particularly those functionalized with R8 and mannose, exhibited excellent physicochemical properties, high CE, and biocompatibility. These nanosystems significantly enhanced mcDNA delivery and both E7mut and E6mut expression in APCs, while increasing the production of pro-inflammatory cytokines, supporting their potential as targeted platforms for DNA vectors against HPV-related cancers. Given the strong performance of the multigenic vector, future studies should prioritize this design to maximize immune responses. Overall, RALA/R8M systems represent a promising advance in the development of efficient and targeted gene delivery strategies for cervical cancer immunotherapy.

## Figures and Tables

**Table 1:** Characterization of mcDNA-E7mut/RALA and mcDNA-E7mut/RALA/R8M NPs. Hydrodynamic diameter, PDI, and zeta potential are presented for each formulation prepared with varying RALA concentrations, while maintaining a fixed mcDNA mass (2 µg) and a fixed R8M concentration (58 µg/mL). NV—non-validated data. No—Formulations without inclusion of R8M; Yes—Formulations with the inclusion of R8M. Two-way ANOVA (within each row, compare columns) was performed for statistical analysis of hydrodynamic diameter, PDI and Zeta potential, followed by post hoc Tukey’s. Statistical difference was present only for hydrodynamic diameter (*p* < 0.05, post hoc Tukey). Different letters indicate significantly different groups.

Formulation	RALA Concentration (µg/mL)	R8M	Hydrodynamic Diameter (nm)	PDI	Zeta Potential (mV)
1	29	No	164.51 ± 21.01 ^a^	0.60 ± 0.10	−5.83 ± 0.18
2	29	Yes	NV	NV	-
3	58	No	172.54 ± 15.91 ^a^	0.44 ± 0.02	−22.10 ± 0.53
4	58	Yes	181.36 ± 9.41 ^a^	0.46 ± 0.03	−19.21 ± 2.75
5	72.5	No	82.72 ± 1.62 ^d^	0.20 ± 0.13	−10.56 ± 3.47
6	72.5	Yes	90.78 ± 10.15 ^d^	0.27 ± 0.04	−6.17 ± 0.24
7	81.2	No	112.16 ± 21.65 ^c^	0.25 ± 0.08	−15.98 ± 7.39
8	81.2	Yes	137.24 ± 22.04 ^b^	0.32 ± 0.05	−13.54 ± 2.71
9	87	No	125.02 ± 6.46 ^c^	0.38 ± 0.02	−23.53 ± 1.33
10	87	Yes	138.91 ± 9.96 ^b^	0.36 ± 0.07	−20.53 ± 1.70
11	116	No	NV	NV	-
12	116	Yes	NV	NV	-

**Table 2:** Characterization of mcDNA/RALA and mcDNA/RALA/R8M NPs formulated with each mcDNA vector (2 µg), RALA (72.5 µg/mL) and R8M (58 µg/mL). Data are presented as mean ± SD from three independent measurements for hydrodynamic diameter, PDI, zeta potential, and CE. No—Formulations without inclusion of R8M; Yes—Formulations with the inclusion of R8M. One-way ANOVA followed by Tukey’s multiple comparisons test showed no statistical differences among the groups.

Formulation	Vector	R8M	Hydrodynamic Diameter (nm)	PDI	Zeta Potential (mV)	CE (%)
1	mcDNA E7mut	No	86.70 ± 1.98	0.25 ± 0.03	−9.87 ± 0.51	98.5 ± 0.91
2		Yes	122.04 ± 17.39	0.30 ± 0.02	−11.04 ± 3.31	97.9 ± 2.23
3	mcDNA E6mut	No	87.05 ± 8.16	0.23 ± 0.03	−7.49 ± 0.19	99.5 ± 0.51
4		Yes	101.79 ± 17.94	0.25 ± 0.04	−12.35 ± 0.42	99.5 ± 0.10
5	mcDNA E7mut-E6mut	No	106.73 ± 12.07	0.29 ± 0.03	−13.25 ± 2.88	99.4 ± 0.23
6		Yes	122.77 ± 20.54	0.29 ± 0.05	−12.71 ± 3.09	99.2 ± 0.04

## Data Availability

The original contributions presented in this study are included in the article/Supplementary Materials. Further inquiries can be directed to the corresponding author.

## Supplementary Materials

The following supporting information can be downloaded at: https://www.mdpi.com/article/10.3390/vaccines14010018/s1, Figure S1: Minicircle DNA recombination process and parental plasmid vectors used in this study. Figure S2: Schematic representation of the molecular modifications designed to inactivate HPV-16 E6 and E7 oncogenic functions.

## Abbreviations

The following abbreviations are used in this manuscript:

ANOVA	Analysis of Variance
APCs	Antigen Presenting Cells
ATCC	American Type Culture Collection
cDNA	Complementary DNA
CE	Complexation Efficiency
CMV	Cytomegalovirus
CPPs	Cell-Penetrating Peptides
DCs	Dendritic Cells
DLS	Dynamic Light Scattering
DNA	Deoxyribonucleic Acid
dsDNA	Double-Stranded DNA
FBS	Fetal Bovine Serum
FTIR	Fourier Transform Infrared Spectroscopy
FW	Forward
GM-CSF	Granulocyte-Macrophage Colony-Stimulating Factor
HICs	High-Income Countries
HPV	Human Papillomavirus
hrHPV	High-Risk Human Papillomavirus
LB	Luria–Bertani
LMICs	Low And Middle-Income Countries
mcDNA	Minicircle DNA
MEM-α	Minimum Essential Medium Alpha
MHC	Major Histocompatibility Complex
mRNA	Messenger Ribonucleic Acid
NPs	Nanoparticles
N/P	Amine to Phosphate
NTC	No-Template Control
PCR	Polymerase Chain Reaction
PDI	Polydispersity Index
pDNA	Plasmid Deoxyribonucleic Acid
PEI	Poly(Ethyleneimine)
pH	Potential of Hydrogen
pRB	Retinoblastoma Protein
R8	Octa-Arginine
R8M	Octa-Arginine Mannose
RNA	Ribonucleic Acid
RTase	Reverse Transcriptase
RT-PCR	Reverse Transcription Polymerase Chain Reaction
RT-qPCR	Quantitative Reverse Transcription Polymerase Chain Reaction
RV	Reverse
SDS	Sodium Dodecyl Sulphate
TB	Terrific Broth
TEM	Transmission Electron Microscopy
