# Supplementary material for: Development of RALA-Based Mannosylated Nanocarriers for Targeted Delivery of Minicircle DNA Vaccines Encoding HPV-16 Oncogenes
Source: Vaccines (Basel). 2025 Dec 23;14(1):18. doi: 10.3390/vaccines14010018 (PMC12846231; doi:10.3390/vaccines14010018)
Supplement: Supplementary file 1 [file vaccines-14-00018-s001.zip › vaccines-3983944-supplementary.pdf]

Supplementary Materials

Development of RALA-Based Mannosylated Nanocarriers for Targeted Delivery of Minicircle DNA Vaccines Encoding HPV-16 Oncogenes

Andressa Giusti [1], Dalinda Eusébio [1], Matilde Costa [1], Inês Silveira [1], Swati Biswas[2], Diana Costa[1] and Ângela Sousa[1,*]

[1] RISE-Health, Department of Medical Sciences, Faculty of Health Sciences, University of Beira Interior, Av. Infante D. Henrique, 6200-506, Covilhã, Portugal; andressa.giusti@ubi.pt (A.G.); dalinda.eusebio@ubi.pt (D.E.); matilde.costa@ubi.pt (M.C.); ines.m.silveira@ubi.pt (I.S.); dcosta@fcsaude.ubi.pt (D.C.)

[2] Nanomedicine Research Laboratory, Department of Pharmacy, Birla Institute of Technology and Science-Pilani, Hyderabad Campus. Jawahar Nagar, Medchal, Hyderabad 500078, India; swati.biswas@hyderabad.bits-pilani.ac.in (S.B.)

* Correspondence: angela@fcsaude.ubi.pt (A.S.); Tel.: + 351 275 329 052

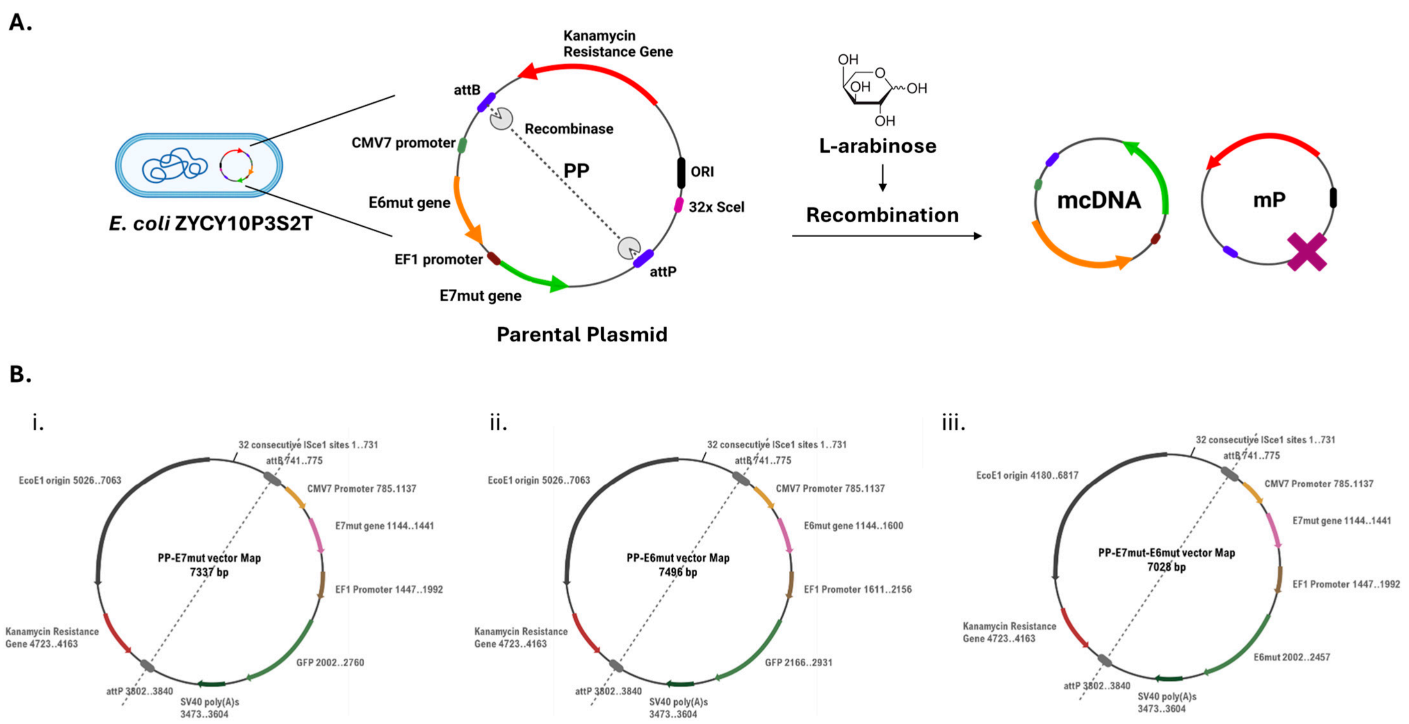

Figure S1: Minicircle DNA recombination process and parental plasmid vectors used in this study. (A) PhiC31/I-SceI L-arabinose–induced recombination of the parental plasmid into a minicircle DNA (eukaryotic sequences) and a miniplasmid (prokaryotic sequences, degraded by enzymes acting on the SceI sites), and (B) the three parental plasmid DNA vectors used in this work: (i) PP-E7mut, (ii) PP-E6mut, and (iii) PP-E7mut-E6mut.

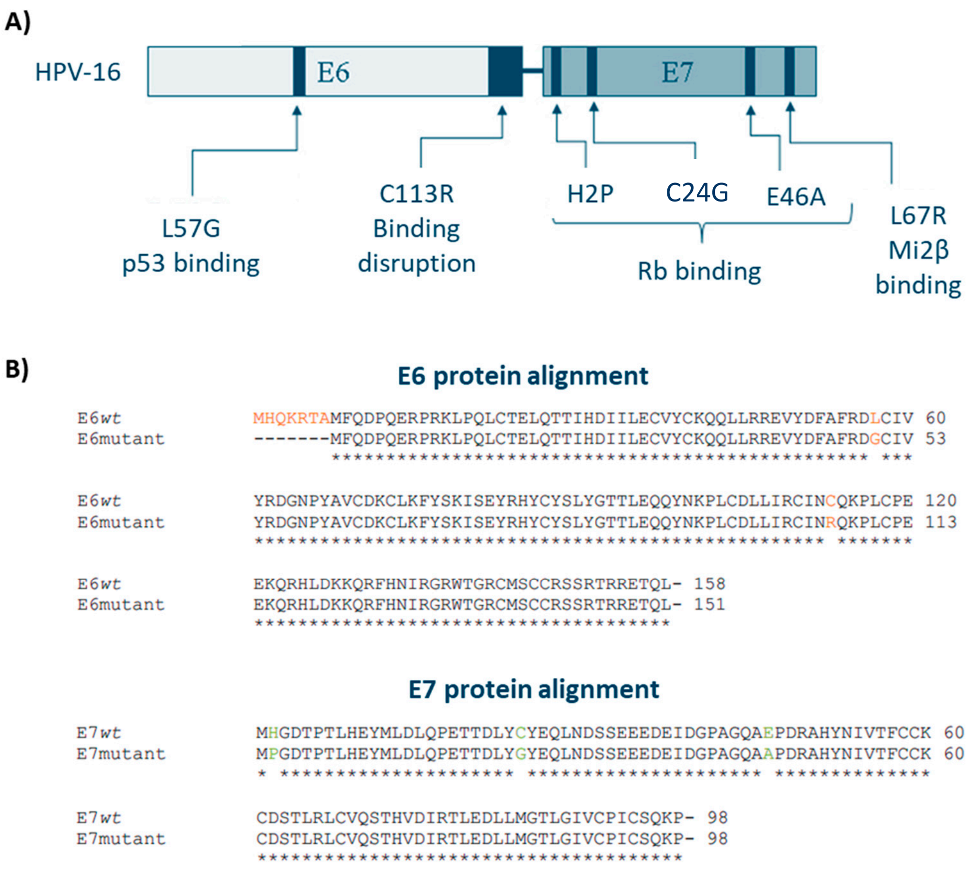

Figure S2: Schematic representation of the molecular modifications designed to inactivate HPV-16 E6 and E7 oncogenic functions. (A) shows the location of mutations, and (B) highlights the corresponding amino acid changes. For E6, L57G impairs p53 degradation and C113R disrupts structural/cofactor interactions. For E7, H2P reduces Rb binding, C24G affects the CXXC motif, E46A alters protein interactions, and L67R disrupts Mi2β binding. Together, these mutations weaken the oncogenic activity of both proteins.
